# Which Is the Best Surgical Approach for Female-to-Male Sexual Reassignment? A Systematic Review of Hysterectomy and Salpingo-Oophorectomy Options from the Gynecological Perspective

**DOI:** 10.3390/medicina60071095

**Published:** 2024-07-04

**Authors:** Mattia Dominoni, Andrea Gritti, Martina Rita Pano, Lucia Sandullo, Rossella Papa, Marco Torella, Barbara Gardella

**Affiliations:** 1 Department of Clinical, Surgical, Diagnostic and Pediatric Sciences, University of Pavia, 27100 Pavia, Italy; andrea.gritti01@universitadipavia.it (A.G.); martinarita.pano01@universitadipavia.it (M.R.P.); b.gardella@smatteo.pv.it (B.G.); 2 Department of Obstetrics and Gynecology, IRCCS Fondazione Policlinico San Matteo, 27100 Pavia, Italy; 3 Obstetrics and Gynecology Unit, Department of Woman, Child and General and Specialized Surgery, University of Campania “Luigi Vanvitelli”, 81100 Naples, Italy; luciasandullo26@gmail.com (L.S.); rossellapapa@gmail.com (R.P.); marcotorella@iol.it (M.T.)

**Keywords:** sexual reassignment, v-NOTES, laparoscopy, robotic surgery, single-site

## Abstract

*Background and Objectives*: Transgender people are defined as individuals whose gender identity does not entirely match their sex assigned at birth. Gender surgery typically represents the conclusive and irreversible step in the therapeutic process, especially for the impact on the reproductive sphere. The increased awareness of gender dysphoria and the expanding array of medical and surgical options, including minimally invasive techniques, contribute to the gradual increase in the social impact of transgender surgery. There are several surgical techniques for “gender assignment”, such as vaginal, laparotomic, laparoscopic, and robotic, and the novel approach of vaginal natural orifice transluminal endoscopic surgery to perform a hysterectomy and bilateral salpingo-oophorectomy (BSO). The purpose of this review is to assess the various surgical approaches (hysterectomy and salpingo-oophorectomy) for gender reassignment in order to determine the best option in clinical practice for the female-to-male population in terms of surgical outcomes such as operative time, surgical complication, hospital discharge, postoperative pain, and bleeding. *Materials and Methods*: This systematic review includes studies from 2007 to 2024. Special consideration was given to articles documenting the characteristics and management of female-to-male reassignment surgery. Finally, eight papers were included in this review. *Results*: The literature analysis considered surgical techniques ranging from traditional surgery to innovative methods like vaginal natural orifice transluminal endoscopic surgery and robotic-assisted laparoscopic hysterectomy. Vaginal natural orifice transluminal endoscopic surgery and the robotic approach offer potential benefits such as reduced postoperative pain and shorter hospital stays. While vaginal natural orifice transluminal endoscopic surgery may encounter challenges due to narrow access and smaller vaginal dimensions, robotic single-site hysterectomy may face instrument conflict. *Conclusions*: The conventional laparoscopic approach remains widely used, demonstrating safety and efficacy. Overall, this review underscores the evolving landscape of surgical techniques for gender affirmation and emphasizes the necessity for personalized approaches to meet the specific needs of transgender patients.

## 1. Introduction

Transgender people are defined as individuals whose gender identity does not entirely match the sex assigned to them at birth. Gender dysphoria is characterized by discomfort due to the disparity between gender identity and the assigned sex at birth [1]. The frequency ranges from 1 in 30.400 to 1 in 200.000, although some recent studies suggest a higher occurrence [2]. For these individuals, psychological counseling is often the first step to facing daily challenges and finding harmony between body and mind [3]. Medical and surgical interventions are justified only if they aim to alleviate or resolve suffering. Effective collaboration across various disciplines becomes crucial for transgender individuals. Gender surgery typically represents the conclusive and irreversible step in the therapeutic process, especially for the impact on the reproductive sphere. Prior to considering a surgical approach, measures like psychotherapy and hormone therapy are implemented to reach the desired phenotypic aspect [4]. Since gender-confirming surgeries can encompass a broad range of demolitive and reconstructive procedures, not all transgender individuals opt for surgery [5]. Surgery is often the final step in addressing gender dysphoria, especially for female-to-male (FTM) patients [6]. The specific surgeries for transitioning from female to male include a subcutaneous mastectomy to create a male-type chest, a hysterectomy or ovariectomy, and a vaginectomy. These procedures can be performed alongside metoidioplasty or phalloplasty, using either a pedunculated or free vascularized flap [7]. The increased awareness of gender dysphoria and the expanding array of medical and surgical options, including minimally invasive techniques, contribute to the gradual increase in the social impact of transgender surgery [4]. There are various surgical techniques for “gender assignment,” such as vaginal, laparotomic, laparoscopic, and robotic, and the novel approach of vaginal natural orifice transluminal endoscopic surgery (v-NOTES) to perform hysterectomy and bilateral salpingo-oophorectomy (BSO). Even if some patients do not desire surgery because they are satisfied only with hormonal therapy, others prefer undergoing surgery to prevent malignancy due to testosterone therapy [8]. In addition, transgender patients may experience emotional distress during a gynecological examination, so surgery represents a possible way to avoid such discomfort [9]. The desire for additional genital surgery such as phalloplasty is even rarer; only a small number of patients actually proceed with it. This kind of surgery has high complication rates and a need for reoperation [10]. That is because the majority of transmen are satisfied with just clitoral hypertrophy and have a fulfilling sexual life, avoiding reconstructive surgery. More frequently, transgender men just ask for hysterectomy or BSO procedures, with the most common request being colpectomy (the removal of vaginal epithelium). Hormonal therapy, hysterectomy alone, and BSO procedures are associated with an increased quality of life for these patients. Some studies have indeed demonstrated that socioeconomic status and mental health are significantly ameliorated by the new patient condition [11].

The purpose of this review is to assess the various surgical approaches (hysterectomy and salpingo-oophorectomy) for gender reassignment in order to determine the best option in clinical practice for the FTM population in terms of surgical outcomes such as operative time, surgical complication, hospital discharge, postoperative pain, and bleeding.

## 2. Materials and Methods

### 2.1. Research Strategy

**Figure 1:**
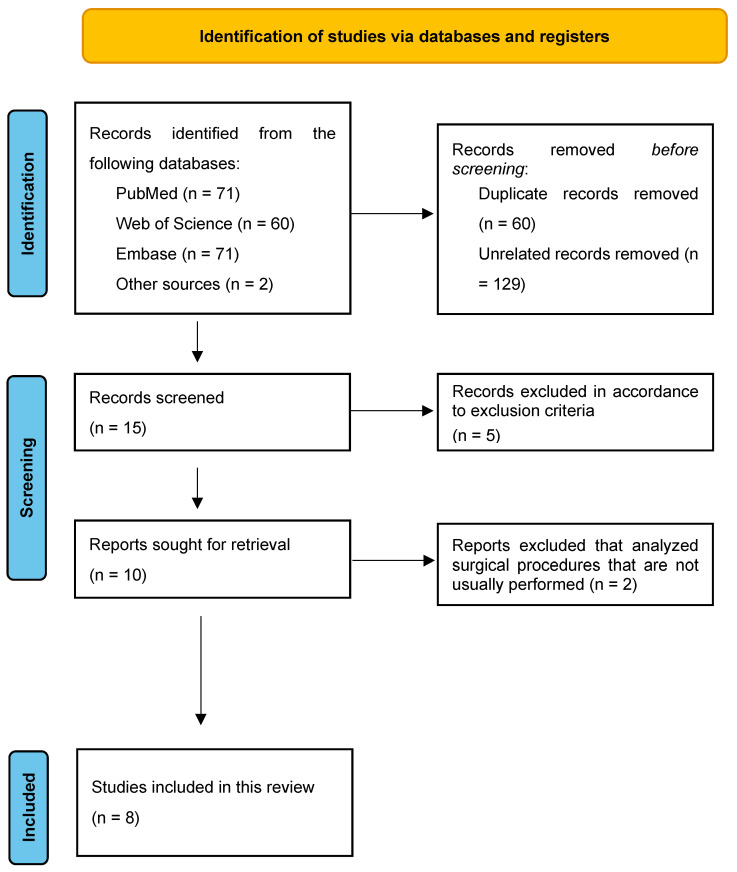
PRISMA Flow chart related to Research strategy.

The Population, Intervention, Control, and Outcome (PICO) design approach was used to divide the main problem into four questions [12,13]. The following keywords combined with the queries reported in Table 1 were used in a literature search considering publications from November 2007 to March 2024, in the PubMed, Web of Science, and Embase databases: “sex reassignment procedures”, “sexual reassignment”, “gender reassignment”, “transgender”, “female-to-male”, “FTM”, “transgender men”, “trans men”, “hysterectomy”, and “salpingo-oophorectomy” along with pluralization, spelling variants between U.S. and U.K. English, and suffixes and prefixes. Using the Preferred Reporting Items for Systematic Reviews and Meta-Analyses (PRISMA) literature selection procedure (Figure 1), we carried out a systematic search [14]. To combine the literature search, two authors independently looked through the reviewed manuscripts’ reference lists. The research strategy is reported in Supplementary S1. This research started on 6 March 2024, and this systematic review concluded on 31 March. This systematic review was registered in PROSPERO on the 3 of May with the number CRD42024538535.

### 2.2. Selection Criteria for Full-Text Article Review

Articles documenting the characteristics and management of female-to-male reassignment surgery were given special consideration. Studies were considered eligible and included in the analysis if they fulfilled the following criteria: (I) female-to-male sexual reassignment procedures; (II) characteristics of sexual reassignment surgery; and (III) surgical management of sexual reassignment surgery. Case series, literature reviews, and prospective and retrospective trials were taken into consideration in this review. The exclusion criteria included the following: single case reports, Conference Proceedings, and Abstracts. In addition, publications written in a language other than English were excluded. We considered articles about the gynecological pertinence of transition excluding urological “reconstructive” surgery. We also excluded studies that analyzed vaginectomy procedures because they are not usually performed during this kind of surgery.

### 2.3. Outcome Measures

The following outcome measures of at least three items (containing three items) had to be reported in the reviewed articles: (I) surgical procedure performed to complete hysterectomy and salpingo-oophorectomy for female-to-male sexual reassignment; (II) blood loss; (III) operative time required to complete the procedures; (IV) hospital stay; (V) postoperative pain using the visual analog scale (VAS); and (VI) the total intra-operative and postoperative adverse events related to each surgical approach.

### 2.4. Risk of Bias

The risk of bias (ROB) of individual studies was assessed by the Revised Cochrane risk-of-bias tool for randomized trials (RoB 2) [15] by two authors (M.R.P. and A.G.) and supervised by two senior authors (M.D. and B.G.). Any disagreements were resolved through discussion with the other authors to reach concordance. Supplementary S2 reports both the graphical and analytical classification of the ROB for each included study.

### 2.5. Data Collection

Two authors verified the data extraction form, and they each extracted data individually (R.P. and L.S.).

### 2.6. Novel Surgical Techniques

While vaginal surgery and laparoscopy have been around for a while, it is worth describing the single-site procedure and v-NOTES surgery to provide the reader with a concept of novel surgical approaches that can also be used for female-to-male sexual reassignment.

Natural orifice transluminal endoscopic surgery (NOTES) is generally employed for several types of procedures and in different settings of patients. It represents a new surgical approach that reassumes and integrates several existing techniques. Specifically, vaginal natural orifice transluminal endoscopic surgery (v-NOTES) can be performed by positioning a v-NOTES port at the introitus, and then a complete hysterectomy and, eventually, salpingo-oophorectomy are carried out transvaginally through endoscopic instruments. This technique is called total vaginal NOTES hysterectomy (TVNH). The v-NOTES port may be created from a size eight nonpowdered sterile glove, a size 50 pessary, and three trocars of 5 mm. A circumferential colpotomy is performed, and the pelvic cavity is reached [16]. Alternatively, an Alexis wound retractor may be positioned, after colpotomy, between the deeper part of the vagina and the perineum, where it is used to induce a pneumoperitoneum and to seal a surgical glove in which trocars may be inserted20. However, nowadays, commercial ports are also available [17]. The endoscope is put through one of the holes in the fingertips of the glove previously created, and it consents visualization of the anatomical structures, while other specific instruments are used to seal and cut the uterosacral ligaments, bilateral cardinal ligaments, transverse cervical fascia, uterine vessels, and ligamentum ovarii. Finally, the uterus is completely detached, and the surgeon proceeds to remove the adnexal structures. All specimens are pulled out through the vagina, which is finally closed forming the vaginal cuff [16,18].

Another technique to perform hysterectomy and bilateral salpingo-oophorectomy is represented by a robotic single-site procedure. After positioning the uterine manipulation device, a first incision at the umbilical level allows for single-site port placement [19]. This is made up of a target anatomy arrow indicator, a room for 4 cannulae, and an insufflation valve. Two of the four cannulae are curved cannulae, whereas two are straight (one for the endoscope and one for the bedside assistant surgeon port). Then, pneumoperitoneum is achieved, and the patient is positioned in the Trendelenburg position, while the da Vinci robot is appropriately situated. A trocar for the robotic endoscope and two trocars for the robotic instruments are positioned in a triangular way. In particular, the monopolar cautery is placed in the second arm, the curved scissor in the first arm, and an assistant’s accessory cannula is used as access for suction/irrigator, for a multifunctional versatile laparoscopic device. Surgical steps consist of transection of the round ligament and incision of the retroperitoneum up to retroperitoneal spaces. Then, ureters are closed off, and the ovarian pedicles are resected. Further steps consist of a total hysterectomy and the removal of the uterus and adnexa through the vagina. Lastly, the vaginal cuff and access port are closed [19]. Generally, the robotic single-port hysterectomy causes only a single scar because it uses a single transumbilical entry point. The robotic multiport technique differs because it has four accesses. Particularly, a Veress needle is inserted to insufflate CO_2_ and to create pneumoperitoneum. Then, four different trocars are put in the abdominal cavity, one in the umbilical, two positioned about 10 cm from the optical trocar on the transverse umbilical line, and the last positioned between the optical trocar and the right hypochondrium [20].

### 2.7. Statistical Analysis

Categorical data are provided as counts and percentages, while continuous variables are provided as the mean ± standard deviation or as the median (interquartile range [IQR]).

## 3. Results

We collected 204 papers from the preliminary bibliographic search, as reported in Figure 1. A total of 15 articles were found after eliminating duplicated papers and unrelated records (Figure 1).

Five articles were excluded since they were outside the aim of this study, dealing with urological reconstructive procedures. Two additional studies were excluded because they described surgical interventions not usually performed after hysterectomy and salpingo-oophorectomy (i.e., colpectomy), given the potentially serious complications (extensive blood loss, vescico-vaginal, or recto-vaginal fistula). We finally considered eight articles for this review. The results of this research are summarized in Table 2 and Table 3, where the most significant results of the articles are reported.

### 3.1. Population

Eight retrospective cohort studies published between 2007 and 2024 were included. A total of 425 trans-sexual men who underwent hysterectomy and bilateral salpingo-oophorectomy for sexual reassignment were included in the studies. Regarding geographical distribution, three trials were conducted in Italy [19,20,21], one in Serbia [23], two in the USA [22,24], one in Turkey [16], and one in Taiwan [18]. Table 2 also reports the demographic characteristics of patients enrolled.

### 3.2. Intervention

Two studies [18,21] reported the application of robotic single-site assisted laparoscopy for hysterectomy and salpigo-oophorectomy in 66 transgender men (TM) (15.52%). One study [20] evaluated the application of multiport-robotic-assisted laparoscopy for sexual reassignment procedures in 20 TM (4.7%). Total laparoscopic hysterectomy and salpingo-oophorectomy were investigated in four studies [16,22,23,24] for a total of 149 subjects (35.05%), while vaginal hysterectomy and salpingo-oophorectomy were performed in three trials [18,22,23] in 142 (33.41) TM. Considering the Obedin-Maliver study [22], 13 out of 14 subjects underwent bilateral salpingo-oophorectomy, while only 1 subject underwent adnexal surgery. Finally, v-NOTES was performed in 35 TMs (8.23%) in two studies [16,18]. In only one paper, abdominal hysterectomy and salpingo-oophorectomy were performed for the surgical procedure of sexual reassignment in 11 TM (2.58%) [22].

### 3.3. Comparison

No randomized clinical trial about the different surgical techniques applied to sexual reassignment surgery is available.

### 3.4. Outcomes

Regarding operative time, robotic single-site procedures required a mean of 140.38 min for hysterectomy and salpingo-oophorectomy [19,21] while multiport robotic surgery required a median of 90 min [20]. In addition, the console time for robotic single-site hysterectomy (RSSH) had a mean of 89.91 min. Docking time required a mean of 8.36 min for a single-site procedure and a median of 15 min for robotic hysterectomy (RH). Three papers [22,23,24] reported a mean of 75.39 min for traditional total laparoscopic surgery (TLH and BSO), and one paper did not report the operative time in minutes [22]. Operative time was not reported for abdominal procedures [22]. Finally, considering vaginal approaches, traditional vaginal surgery required a mean of 100.2 min [18,22,23], but one trial did not analyze operative time. On the other hand, two studies [16,18] reported operative time for v-NOTES (mean of 270.4 min).

Analyzing blood loss, in one study, a robotic single-site procedure reported a mean of 30 mL, and a mean Hb drop of 1.1 g/dL was reported in another study [19,21]. Multiport robotic surgery had a median of 90 mL [20]. One paper [22] reported a median of 175 mL and another paper [24] reported a mean of 26.88 mL for traditional laparoscopic surgery; in addition, one paper [16] reported a mean Hb drop of 1.5. One paper did not report blood loss [23]. One paper reported a median blood loss of 225 mL for abdominal procedures [22]. Finally, considering vaginal surgery, two articles [18,23] reported a mean blood loss of 200 mL for the traditional vaginal approach, while one paper did not report the value of blood loss. Two studies [16,18] reported blood loss for v-NOTES as follows: a median of 200 mL in one study [18], and a mean Hb drop of 1.5 in the other paper [16].

Two studies [19,21] reported a hospitalization of 3.15 days for the robotic single-site approach, while RH had a median of 2.5 days [20]. Hospital stay was not reported for the abdominal approach [22]. Three studies [16,23,24] reported a mean hospitalization of 2.65 days for laparoscopic surgery, while one paper did not report any data [22]. Two papers [18,23] reported a mean of 5.55 days for transvaginal surgery, and one paper did not analyze hospitalization [22]. In the case of v-NOTES, the mean was 4.65 days, as reported in two trials [16,18]. In overall vaginal approaches, there were several discrepancies among the values reported.

Three papers did not report any data about the visual analog scale (VAS) in postoperative time [22,23,24]. Analyzing the values of the VAS in the papers considered, there were differences in the time of VAS measurement in all the works reported in Table 2. Analyzing the general trend in the VAS, it was clear that in all the surgical procedures, there was a decrease in postoperative pain.

Finally, considering the overall complications (intra-operative and postoperative), the results were the following: two papers [19,21] reported a mean of two complications for RSSH, while one paper [20] did not report if any complications occurred for RH. For the laparoscopic group, the mean number of complications was two in three papers [16,23,24], while for the vaginal procedure, it was three in two papers [18,22,23]. It is important to mention that in one paper [22], the number of complications was not differentiated among TLH, vaginal hysterectomy (VH), and abdominal hysterectomy (AH); for this reason, it was not computed in the previous mean evaluation. Finally, considering v-NOTES, the mean number of complications was 0.5 (in two papers) [16,18].

## 4. Discussion

This study highlights the use of various surgical techniques, including laparoscopic, robotic-assisted, transvaginal, and v-NOTES (which can be total or vaginal-assisted) hysterectomy and salpingo-oophorectomy. Surgical procedures vary from traditional approaches such as laparotomy, laparoscopy, or the vaginal route to more recent approaches like v-NOTES or robotic surgery. Prior research has examined how gender affirmation surgery affects the psycho-social and sexual functioning of transgender subjects. However, little is known about the prevalence of hysterectomy and related perioperative adverse events in transgender patients pursuing gender affirmation treatment. In the TM population, Bretschneider’s cross-sectional population-based survey reported that laparoscopic hysterectomy was the most common procedure performed (57.2%), followed by laparoscopic-assisted vaginal (20%), abdominal (15.2%), and vaginal hysterectomy (7.7%) [25].

The inclusion of novel techniques such as v-NOTES and robotic-assisted single-site laparoscopic hysterectomy reflects advancements in minimally invasive surgery, offering potential benefits such as reduced postoperative pain and shorter hospital stays. Both appear to be indicated for patients undergoing female-to-male transition surgery.

Regarding the main outcomes of this review, operation time in trans-sexual subjects who underwent v-NOTES and the vaginal approach was greater with respect to laparoscopy; in addition, also in the case of robotic surgery, the operation time was higher than in the case of laparoscopy. On the other hand, blood loss appeared to be higher in vaginal surgery (VH and v-NOTES) than in the laparoscopic approach (traditional laparoscopy and robotic surgery). As Gardella et al. suggested for the TM group, the absence of adhesions at the abdominal wall and viscera may be an important factor in reducing operative times. In addition, TM patients had a smaller uterus, which simplified the dissection of the anatomical planes. On the contrary, the extraction of surgical specimens may be more difficult because of the atrophy of the vaginal wall and the narrow space [21]. The differences in total operative time are probably derived from the different skills of surgeons, the comorbidity of patients, and the time request for instrument organization, especially in the case of robotic surgery and v-NOTES. v-NOTES offers a minimally invasive approach with no visible scars, but it may be challenging because of limited access and smaller vaginal dimensions and atrophy in transgender male patients, mainly due to the use of androgen-based therapies. The most critical factor is the conflict among instruments, given the narrow route of access. This can be avoided using proper endoscope selection. The trans-sexual male vagina is often smaller because of virginity and nulliparity, and it is atrophic because of hormonal therapy [19]. However, trans-sexual males tend to have smaller uterine weight, length, width, and depth [24]. In addition, the v-NOTES technique represents a more feasible route to perform BSO compared with traditional vaginal procedures when removing ovaries regardless of their position in the pelvic cavity and even in the presence of adhesions.

Concerning the single-site approach, BMI had no negative impact on surgical outcomes, and obesity did not appear to be a barrier to the single-site strategy. In contrast to other traditional types of surgery, age and BMI did not appear to influence the operating length or outcomes of patients undergoing RSSH. Gupta et al. found that younger individuals with a lower BMI were more likely to have standard laparoscopy or RSSH [26]. The single-site approach necessitates surgical experience because the movements are forced and vision is limited. The literature data show that the reduction in operative time is correlated to surgeon experience, and surgical skill influences all operative times, regardless of the complexity of surgical cases [26].

Nowadays, the introduction of vaginal surgery has resulted in a longer hospital stay than both laparoscopic and robotic surgery, with the latter having the shortest value. On the other hand, laparoscopic, robotic, and conventional vaginal surgery are procedures associated with the highest average number of problems after v-NOTES surgery. On the other hand, robotic surgery is an expensive surgical procedure, but Bogliolo et al. showed that, for benign indications, the robotic single-site technique is less expensive than the multiport approach, saving between USD 1500 and USD 2000 for each single-site procedure [27].

Because the articles’ various times of consideration prevented a comparison, our review’s assessment of postoperative pain using the VAS was not comprehensive. Nevertheless, research has shown that FTM tend to have lower VAS scores following surgery compared with laparoscopy, and they also tend to report reduced postoperative pain when using the v-NOTES approach. Assessing the complication rate, no difference was found between the two groups [16]. Additionally, comparing VH and v-NOTES, the latter is associated with less postoperative pain [18].

Regarding the possible benefits of minimally invasive surgery in FTM, particular attention was paid to scar-less surgery, especially considering the psychological well-being of TM. Furthermore, patients who underwent single-site surgery reported a considerable improvement in their well-being because the operation had less of an impact on their view of their bodies, as well as a large rise in personal satisfaction with the quality of the surgical scar. Not only does the presence of a single scar improve cosmetic results, but it may reduce the risk of inferior epigastric or circumflex ileac artery transection, which is important in eventual reconstructive surgery [19,28]. The multiport robotic technique constitutes a viable alternative surgery for TM patients for several reasons such as better triangulation and a higher range of movements because of the greater distance between laparoscopic accesses; however, it does not obtain the same cosmetic result as single-port surgery because of the greater number of accesses, which are also causes of major pain and recognizable signs of surgery [20]. Nowadays, v-NOTES is a possible scar-less approach that may reduce the need to resort to abdominal surgery, preserve the integrity of the abdominal wall, avoid useless scars, and ensure a good cosmetic result. This surgical technique combines the benefits of laparoscopic surgery and the cosmetic outcomes, using the advantages of vaginal surgery [28]. As surgical options continue to evolve, personalized approaches adapted to individual needs will be crucial in optimizing outcomes and improving the overall quality of care for transgender patients undergoing gender reassignment surgeries.

It is of paramount importance that surgeons have significant experience with traditional laparoscopy in order to navigate the learning curve for laparoscopy; nevertheless, the robotic technique appears to be easier because of its enhanced dexterity, wristed equipment, and enlarged three-dimensional vision. For this reason, younger surgeons can manage the learning curve associated with the robotic single-site method. Furthermore, from a future perspective, young surgeons may find the application of the vaginal approach to be more common from a minimally invasive standpoint by using the v-NOTES technique in conjunction with a minimally invasive laparoscopic approach.

In addition, it is interesting to evaluate the minimally invasive approach proposed for TM also in cisgender women (CW), in order to evaluate the possible difference or benefit between the laparoscopic and vaginal routes. In our previous study [21], we compared single-site and multiport robotic surgery in TM and CW. The surgical times for TM and CW differed statistically (total operative time = 0.0152, docking time = 0.0011, console time = 0.0001, anesthetic time = 0.0061). There was also a significant difference in the body mass index (*p* = 0.0169), uterine volume (*p* = 0.0001), and prior comorbidity (*p* = 0.0001) aside from TM. The two groups did not differ in terms of conversion rate, reduction in hemoglobin and blood loss, length of hospital stay, or intra- and postoperative problems. When compared with other purposes for benign disease, RSSH for sex reassignment looks to be a safe, practical, and affordable choice with a significant reduction in surgery time. Furthermore, it seems that the advantages of this scar-lees surgical technique are more noticeable in TM. Similarly, Chen et al. reported that TM had a much shorter operating duration than CW with menstrual problems, although they used a different surgical technique [29].

Analyzing the traditional laparoscopic approach between TM and CW, O’Hanlan et al. [24] reported that the operating times for TM surgeries were shorter (mean 74 min versus 120 min, median 57.5 min versus 116 min, *p* < 0.001), there was less blood loss (mean 27 mL versus 107 mL, median 20 mL versus 50 mL, *p* < 0.001), and the uterine weight was lower (mean 118 g versus 167 g, median 89 g versus 140.5 g, *p* < 0.001). Both the overall (12.2% vs. 8.3%) and re-operative (4.9% vs. 4.3%) complication rates did not differ statistically from one another. Because of this, patients who identify as trans-sexual can have satisfactory surgical outcomes with a total laparoscopic hysterectomy.

In addition, analyzing hysterectomies performed by the traditional vaginal route or by v-NOTES in CW, Merlier et al. [30] reported that v-NOTES can be carried out as a suitable and safe substitute for VH. Except for the rate of salpingectomies or adnexectomies, which was significantly higher in the v-NOTES group (with 100% of patients undergoing one of these procedures compared with 60% in the vaginal route group; *p* < 0.001), there was no difference in the surgical outcomes between the two groups in CW. To our knowledge, no paper has reported a comparison between TM and CW with the v-NOTES approach. On the other hand, a previous paper compared the vaginal route for hysterectomy in TM and CW. In total, 42% of cisgender women and 24% of transgender men had vaginal hysterectomies. The estimated blood loss was lower in TM (P5.002), but the difference between the gender groups was no longer significant when uterine size and hysterectomy route were taken into account. The number of patients reporting problems did not differ between the groups. Vaginal hysterectomy should be taken into consideration when planning surgery for TM because it is a feasible operation for this demographic, as the authors of [22] attested.

Finally, the surgical approaches involved in FTM sexual reassignment may involve pelvic floor structures. Nowadays, the literature data about the role of surgery in the risk of developing pelvic floor dysfunction in TM is poor and not complete or decisive. Pelvic floor dysfunction is becoming a more significant problem in transgender persons. This increasing prevalence could be caused by a variety of factors, including comorbidities, socioeconomic level, and hormone medication, which may have an effect on the organization of connective and adipose tissue, pelvic organ support systems, and pelvic floor muscle mass. Surgery for sexual reassignment may affect the pelvic floor and result in pelvic floor dysfunction, which may lead to prolapse of the pelvic organs, urine incontinence, problems with sexual pleasure, or problems with overall wellness [25,31,32]. Furthermore, androgen therapy produces epithelial thinning and suppresses estrogens in a manner similar to that of estrogen deprivation. This leads to anatomical changes in the vulvar, urethral, vaginal wall, and bladder tissues, which cause genital dryness, friable vaginal epithelium, frequent urination, and urgency [33,34].

### Limitations and Strengths

The main limitation of this study was the small number of articles selected. In addition, the analyzed data were derived from retrospective works, and a comparison was difficult because of the different measurement and analysis methods. However, the number of transgender male patients who undergo hysterectomy and BSO is now increasing, which will lead to a greater number of studies available in the future. Another potential drawback was the inability to take into account the lifestyle choices, hormonal therapy, and demographic traits that could play a major role in determining the surgical strategy. Furthermore, this review did not address the psychological effects of surgery or the aesthetic result for the TM who underwent it.

This research was the first to examine the potential surgical options for gender reassignment surgery in terms of surgical results and comorbidities. This analysis provides us with a broad overview of the options that are currently available to ensure that the FTM population has a customized and trustworthy approach that is based on the unique characteristics of each subject.

## 5. Conclusions

This review highlights the different gynecological surgical techniques available for sexual reassignment procedures, from traditional approaches to innovative methods like v-NOTES and robotic-assisted procedures. This review clarifies that laparoscopic surgery results in shorter operating times, less blood loss, and shorter hospital stays. Nonetheless, the risk of surgical complications is significantly decreased by vaginal surgery, particularly v-NOTES. In order to ensure excellent surgical success, high quality of life, and psycho-social well-being, new techniques like v-NOTES in gender reassignment surgery will likely enable us to combine the advantages of the laparoscopic technique with the benefits of vaginal surgery and an aesthetic outcome.

## Figures and Tables

**Table 1:** Research queries according to PICO criteria.

Query	Population	Intervention	Comparison	Outcomes
1	female-to-male	Hysterectomy and salpingo-oophorectomy	none	v-NOTES/vaginal surgery reduces operative time
2	female-to-male	Hysterectomy and salpingo-oophorectomy	none	Laparoscopic/robotic surgery reduce operative time
3	female-to-male	Hysterectomy and salpingo-oophorectomy	none	v-NOTES/vaginal surgery reduces intra and postoperative complications
4	female-to-male	Hysterectomy and salpingo-oophorectomy	none	Laparoscopic/robotic surgery reduces intra- and postoperative complications
5	female-to-male	Hysterectomy and salpingo-oophorectomy	none	v-NOTES/vaginal surgery reduces postoperative pain
6	female-to-male	Hysterectomy and salpingo-oophorectomy	none	Laparoscopic/robotic surgery reduces postoperative pain
7	female-to-male	Hysterectomy and salpingo-oophorectomy	none	v-NOTES/vaginal surgery reduces hospital stay
8	female-to male	Hysterectomy and salpingo-oophorectomy	none	Laparoscopic/robotic surgery reduces hospital stay

**Table 2:** Demographic characteristics of the enrolled patients.

Study and Year	Origin of Study	Patients Enrolled	Age (Mean ± SD)	BMI (Mean ± SD)	Parity (N, %)	Uterine Weight (Mean ± SD) g
Bogliolo et al., 2014 [19]	Italy	10 TM	28 ± 5.7	22 ± 1.7	0	Not reported
Gardella et al., 2021 [21]	Italy	60 TM	30.62 ± 7.93	23.52 ± 4.26	0	Nor reported
Giampaolino et al., 2021 [20]	Italy	20 TM	Median: 23.5 (19.5–28.4)	Median: 22.5 (range: 21–24.7)		Not reported
Obedin-Maliver, 2017 [22]	USA	33 TM	35.2 ± 69.9	27.9 ± 65.4	2 (6.1)	Not reported
Jeftovic et al., 2018 [23]	Serbia	124 TM	Not reported	Not reported	Not reported	Not reported
O’Hanlan et al., 2007 [24]	USA		31.76 ± 7.4	27.36 ± 5.8	2 (4.9)	118.02 ± 115.6
Donmez et al., 2024 [16]	Turkey	83 TM	v-NOTES: 27.57 ± 3.9 TLH: 26.6 ± 4.8	v-NOTES: 22.9 ± 2.8 TLH: 24.8 ± 4.4	Not reported	Not reported
Lee et al., 2018 [18]	Taiwan	56 TM	VH: 29.3 ± 6.4 v-NOTES: 28.8 ± 7.3	VH: 23.7 ± 4.4 v-NOTES: 24.3 ± 5.0	VH:0 v-NOTES:0	VH: not reported v-NOTES: not reported

Legend: standard deviation: SD; transgender men: TM; total laparoscopic hysterectomy: TLH; vaginal hysterectomy: VH; vaginal natural orifice transluminal endoscopic surgery: v-NOTES.

**Table 3:** Main results of the studies included in this review.

Study and Year	Study Design	Patients Enrolled	Procedure	Operative Time	Blood Loss, mL	Hospital Stay, Days	Visual Analog Scale (VAS)	Intra- and Postoperative Complications, n (%)
Bogliolo et al., 2014 [19]	Retrospective study	10 TM	RSSH and mean ± SD minutes BSO	Mean ± SD minutes Operative time: 137 ± 32. Console time:79 ± 15. Docking time: 9 ± 2 m.	Mean ± SD 30 ± 24 mL	Mean ± SD days 2.4 ± 0.9	Mean VAS: 1 (interquartile 0–3 at 1 h after surgery) VAS: 0 (interquartile 0–0) at 24 h after surgery	1 (10)
Gardella et al., 2021 [21]	Prospective monocentric study	60 TM	RSSH and BSO	Mean ± SD Total surgical time: 143.77 ± 40.39. Console time: 100.73 ± 32.26. Docking time: 7.72 ± 2.61.	Hb drop mean ± SD: 1.1 ± 0.46	3.85 ± 1.26	Mean ± SD VAS: 4.53 ± 1.73 at 1 h after surgery VAS: 2.35 ± 1.96	3 (4.83)
Giampaolino et al., 2021 [20]	Single-center retrospective study	20 TM	RH and BSO	Median Operation time: 90 (interquartile 65–150). Docking time: 15 (interquartile 10–25) minutes. Time spent in the operating room: 140 (90–180).	Median 90 (interquartile 150–30) mL Decrease in hemoglobin levels (%): 8 (4–16)	Median 2.5 (interquartile 2–4)	Median VAS: 5 (interquartile 3–8) score in the immediate postoperative period. VAS: 3 (interquartile 1–6) at 24 h after surgery. VAS: 2 (interquartile 0–5) at 28 h after surgery.	Not reported
Obedin-Maliver, 2017 [22]	Single-center retrospective cohort study	33 TM	14 TM: TLH and 13 BSO (one subject only adnexal surgery). 8 TM: VH and BSO. 11 TM: AH and BSO.	Not reported	Median TLH and BSO:175 (interquartile 110–30). VH and BSO: 250 (interquartile 175–400). AH and BSO: 225 (interquartile 200–250).	Not reported	Not reported	9 (27.3)
Jeftovic et al., 2018 [23]	Retrospective study	124 TM	92 TM: VH and BSO 32 TM: TLH and BSO	Mean VH:51 (interquartile 46–72) TLH: 76 (interquartile 68–90)	Not reported	Mean VH: 4 (interquartile 3–6) TLH 4 (interquartile 3–6)	Not reported	VH: 1 (1%) TLH: 1 (3%)
O’Hanlan et al., 2007 [24]	Retrospective study	41 TM	THL and BSO	Mean ± SD 74.08 ± 35.4	Mean ± SD 26.88 ± 27.7	Mean ± SD 1.07 ± 0.3	Not reported	5 (12.2)
Donmez et al., 2024 [16]	Retrospective cohort study	83 TM	21 TM: v-NOTES and BSO 62 TM: TLH and BSO	mean ± SD: v-NOTES: 126.1 ± 37.9 TLH: 76.1 ± 33.9	Hemoglobin drop, mean ± SD v-NOTES: 1.5 ± 0.9 In TLH: 1.5 ± 0.9 (0.1–3.4)	Postoperative hospital stay, days: v-NOTES: 1.6 ± 1.01 TLH: 2.9 ± 0.5	Postoperative pain second hour after surgery, median ± SD v-NOTES: 5 ± 1.56 TLH: 8 ± 1.11 Postoperative 24th hour after surgery, median ± SD TVNH: 1 ± 0.62 TLH: 2 ± 0.9	0
Lee et al., 2018 [18]	Retrospective study	56 TM	42 TM: VH and BSO 14: v-NOTES and BSO	mean ± SD: v-NOTES: 144.3 ± 51.7 VH: 149.2 ± 47.1	median v-NOTES: 200 (interquartile 100–388) VH:150 mL (interquartile 100–350)	mean ± SD v-NOTES: 7.7 ± 2.4 VH: 7.1 ± 3.1	VAS at 2 h and 72 h, mean ± SD: v-NOTES: 4.9 ± 3.0 and 1.7 ± 1.0 (n = 12) VH: 7.1 ± 1.4 (n = 42) and 2.7 ± 1.1 (n = 34)	v-NOTES 1 (0.025) VH: 5 (11.9)

Legend: standard deviation: SD; robotic-assisted single-site laparoscopic hysterectomy: RSSH; multiport robotic-assisted laparoscopic hysterectomy: RH; abdominal hysterectomy: AH; bilateral salpingo-oophorectomy: BSO; transgender men: TM; total laparoscopic hysterectomy: TLH; vaginal hysterectomy: VH; vaginal natural orifice transluminal endoscopic surgery: v-NOTES; VAS: visual analog scale; Hb: hemoglobin.

## Data Availability

Not applicable.

## Supplementary Materials

The following supporting information can be downloaded at: https://www.mdpi.com/article/10.3390/medicina60071095/s1, S1: Research strategy link. S2: Risk of bias in the included studies.

## References

[1] Davis L.C., Diianni A.T., Drumheller S.R., Elansary N.N., D’ambrozio G.N., Herrawi F., Piper B.J., Cosgrove L. (2024). Undisclosed financial conflicts of interest in DSM-5-TR: Cross sectional analysis. BMJ.

[2] Meier S.C., Labuski C.M., Baumle A.K. (2013). The demographics of the transgender population. International Handbook on the Demography of Sexuality.

[3] Ghiasi Z., Khazaei F., Khosravi M., Rezaee N. (2024). Physical and psychosocial challenges of people with gender dysphoria: A content analysis study. BMC Public Health.

[4] Coleman E., Radix A.E., Bouman W.P., Brown G.R., de Vries A.L.C., Deutsch M.B., Ettner R., Fraser L., Goodman M., Green J. (2022). Standards of Care for the Health of Transgender and Gender Diverse People, Version 8. Int. J. Transgender Health.

[5] Schechter L.S. (2016). Gender Confirmation Surgery: An Update for the Primary Care Provider. Transgender Health.

[6] Manrique O.J.M., Bustos S.S., Bustos V.P., Mascaro A.A., Ciudad P., Forte A.J., Del Corral G., Kim E.A.M., Langstein H.N. (2021). Building a Multidisciplinary Academic Surgical Gender-affirmation Program: Lessons Learned. Plast. Reconstr. Surg.—Glob. Open.

[7] Monstrey S., Ceulemans P., Hoebeke P. (2011). Sex Reassignment Surgery in the female-to male Transsexual. Semin. Plast. Surg..

[8] Braun H., Nash R., Tangpricha V., Brockman J., Ward K., Goodman M. (2017). Cancer in Transgender People: Evidence and Methodological Considerations. Epidemiol. Rev..

[9] Dutton L., Koenig K., Fennie K. (2008). Gynecologic Care of the Female-to-Male Transgender Man. J. Midwifery Womens Health.

[10] Kang A., Aizen J.M., Cohen A.J., Bales G.T., Pariser J.J. (2019). Techniques and considerations of prosthetic surgery after phalloplasty in the transgender male. Transl. Androl. Urol..

[11] Kilmer L.H., Chou J., Campbell C.A., DeGeorge B.R., Stranix J.T. (2023). Gender-Affirming Surgery Improves Mental Health Outcomes and Decreases Anti-Depressant Use in Patients with Gender Dysphoria. Plast. Reconstr. Surg..

[12] Methley A.M., Campbell S., Chew-Graham C., McNally R., Cheraghi-Sohi S. (2014). PICO, PICOS and SPIDER: A comparison study of specifcity and sensitivity in three search tools for qualitative systematic reviews. BMC Health Serv. Res..

[13] Aslam S., Emmanuel P. (2010). Formulating a researchable question: A critical step for facilitating good clinical research. Indian J. Sex. Transm. Dis. AIDS.

[14] Moher D., Liberati A., Tetzlaff J., Altman D.G. (2009). Preferred Reporting Items for Systematic Reviews and Meta-Analyses: The PRISMA Statement. J. Clin. Epidemiol..

[15] Higgins J.P., Altman D.G., Gøtzsche P.C., Jüni P., Moher D., Oxman A.D., Savovic J., Schulz K.F., Weeks L., Sterne J.A. (2011). The Cochrane Collaboration’s tool for assessing risk of bias in randomised trials. BMJ.

[16] Donmez E.E., Elci E., Elci G. (2024). Total vNOTES hysterectomy versus conventional total laparoscopic hysterectomy in virgin transgender men. Minim. Invasive Ther. Allied Technol..

[17] Lerner V.T., May G., Iglesia C.B. (2023). Vaginal Natural Orifice Transluminal Endoscopic Surgery Revolution: The Next Frontier in Gynecologic Minimally Invasive Surgery. JSLS.

[18] Lee Y.L., Hsu T.F., Jiang L.Y., Chao H.T., Wang P.H., Chen Y.J. (2019). Transvaginal Natural Orifice Transluminal Endoscopic Surgery for female-to male Transgender Men. J. Minim. Invasive Gynecol..

[19] Bogliolo S., Cassani C., Babilonti L., Gardella B., Zanellini F., Dominoni M., Santamaria V., Nappi R.E., Spinillo A. (2014). Robotic Single-Site Surgery for female-to male Transsexuals: Preliminary Experience. Sci. World J..

[20] Giampaolino P., Della Corte L., Improda F.P., Perna L., Granata M., Sardo A.D.S., Bifulco G. (2021). Robotic Hysterectomy as a Step of Gender Affirmative Surgery in female-to male Patients. J. Investig. Surg..

[21] Gardella B., Dominoni M., Bogliolo S., Spinillo A. (2021). Surgical outcome for robotic-assisted single-site hysterectomy (RSSH) in female-to male reassignment compared to its use in benign gynecological disease: A single center experience. J. Robot. Surg..

[22] Obedin-Maliver J., Light A., De Haan G., Jackson R.A. (2017). Feasibility of Vaginal Hysterectomy for female-to male Transgender Men. Obstet. Gynecol..

[23] Jeftovic M., Stojanovic B., Bizic M., Stanojevic D., Kisic J., Bencic M., Djordjevic M.L. (2018). Hysterectomy with Bilateral Salpingo-Oophorectomy in female-to male Gender Affirmation Surgery: Comparison of Two Methods. BioMed Res. Int..

[24] O’Hanlan K.A., Dibble S.L., Young-Spint M. (2007). Total Laparoscopic Hysterectomy for female-to male Transsexuals. Obstet. Gynecol..

[25] Bretschneider C.E., Sheyn D., Pollard R., Ferrando C.A. (2018). Complication rates and outcomes after hysterectomy in transgender men. Obstet. Gynecol..

[26] Gupta N., Blevins M., Holcombe J., Furr R.S. (2020). A Comparison of Surgical Outcomes between Single-Site Robotic, Multiport Robotic and Conventional Laparoscopic Techniques in Performing Hysterectomy for Benign Indications. Gynecol. Minim. Invasive Ther..

[27] Bogliolo S., Ferrero S., Cassani C., Musacchi V., Zanellini F., Dominoni M., Spinillo A., Gardella B. (2016). Single-site versus multiport robotic hysterectomy in benign gynecologic diseases: A retrospective evaluation of surgical outcomes and cost analysis. J. Minim. Invasive Gynecol..

[28] Jallad K., Siff L., Thomas T., Paraiso M.F.R. (2016). Salpingo-Oophorectomy by Transvaginal Natural Orifice Transluminal Endoscopic Surgery. Obstet. Gynecol..

[29] Chen I., Nguyen V., Hodge M., Mallick R., Gagné H., Singh S.S., Choudhry A.J., Xie R., Liao Y., Wen S.W. (2020). Surgical outcomes for transgender men undergoing hysterectomy. J. Obstet. Gynaecol. Can..

[30] Merlier M., Collinet P., Pierache A., Vandendriessche D., Delporte V., Rubod C., Cosson M., Giraudet G. (2022). Is V-NOTES Hysterectomy as Safe and Feasible as Outpatient Surgery Compared with Vaginal Hysterectomy?. J. Minim. Invasive Gynecol..

[31] Melloni C., Melloni G., Rossi M., Rolle L., Carmisciano M., Timpano M., Falcone M., Frea B., Cordova A. (2016). Lower Urinary Tract Symptoms in Male-to-Female Transsexuals: Short Terms Results and Proposal of a New Questionnaire. Plast. Reconstr. Surg. Glob. Open.

[32] Combaz N., Kuhn A. (2017). Long-Term Urogynecological Complications after Sex Reassignment Surgery in Transsexual Patients: A Retrospective Study of 44 Patients and Diagnostic Algorithm Proposal. Am. J. Urol. Res..

[33] Krakowsky Y., Potter E., Hallarn J., Monari B., Wilcox H., Bauer G., Ravel J., Prodger J.L. (2022). The effect of gender-affirming medical care on the vaginal and neovaginal microbiomes of transgender and gender-diverse people. Front. Cell. Infect. Microbiol..

[34] Clark A.L., Goetsch M.F. (2024). Genitourinary syndrome of menopause: Pathophysiology, clinical presentation, and diferential diagnosis. Clin. Obstet. Gynecol..

