# Supplementary material for: Which Is the Best Surgical Approach for Female-to-Male Sexual Reassignment? A Systematic Review of Hysterectomy and Salpingo-Oophorectomy Options from the Gynecological Perspective
Source: Medicina (Kaunas). 2024 Jul 4;60(7):1095. doi: 10.3390/medicina60071095 (PMC11278962; doi:10.3390/medicina60071095)
Supplement: Supplementary file 1 [file medicina-60-01095-s001.zip › medicina-3041145-supplementary.pdf]

**S1: search strategy**

**PubMed:**

("Sex Reassignment Procedures"[Mesh] OR "Sexual Reassignment" OR "Gender Reassignment" OR "Transgender") AND ("Female-to-Male" OR "FTM" OR "Transgender Men" OR "Trans Men") AND ("Hysterectomy"[Mesh] OR "Hysterectomy" OR "Salpingo-Oophorectomy"[Mesh] OR "Salpingo-Oophorectomy")

**EMBASE**

('gender reassignment'/exp OR 'sexual reassignment':ab,ti OR 'gender reassignment':ab,ti OR 'transgender':ab,ti) AND ('female-to-male':ab,ti OR 'ftm':ab,ti OR 'transgender men:ab,ti' OR 'trans men':ab,ti) AND ('hysterectomy'/exp OR 'hysterectomy':ab,ti OR 'salpingooophorectomy'/exp OR 'salpingo-oophorectomy':ab,ti) AND ('article'/it OR 'review'/it)

**WEB OF SCIENCE**

(“sex reassignment” OR "Sex Reassignment Procedures" OR “gender reassignment” OR transgender) AND (“female to male” OR FTM OR “transgender men” OR “trans men”) AND (hysterectomy OR “salpingo-oophorectomy” OR salpingooophorectomy ) (Topic) AND Article or Review Article or Early Access (Document Types)

S2: Risk of bias evaluation

| Unique ID | Study ID | Experimental | Comparator | Outcome | Weight | D1 | D2 | D3 | D4 | D5 | Overall |
|---|---|---|---|---|---|---|---|---|---|---|---|
| V1 | Bogliolo et al, 2 | NA | NA | NA | 1 | ! | + | + | + | + | ! |
| V2 | Gardella et al, 2 | NA | NA | NA | 1 | ! | + | + | + | + | ! |
| V3 | Giampaolino et | NA | NA | NA | 1 | ! | + | + | + | + | ! |
| V4 | Obedin-Maliver | NA | NA | NA | 1 | ! | + | + | + | + | ! |
| V5 | Jeftovic et al, 20 | NA | NA | NA | 1 | ! | + | + | + | + | ! |
| V6 | O'Hanlan et al, | NA | NA | NA | 1 | ! | + | + | + | + | ! |
| V7 | Donmez et al, 2( | NA | NA | NA | 1 | ! | + | + | + | + | ! |
| V8 | Lee et al, 2018 | NA | NA | NA | 1 | ! | + | + | + | + | ! |

| | |
|---|---|
| + | Low risk |
| ! | Some concerns |
| - | High risk |
| D1 | Randomisation process |
| D2 | Deviations from the intended interventions |
| D3 | Missing outcome data |
| D4 | Measurement of the outcome |
| D5 | Selection of the reported result |
